# Safe and effective aerosolization of in vitro transcribed mRNA to the respiratory tract epithelium of horses without a transfection agent

**DOI:** 10.1038/s41598-020-79855-1

**Published:** 2021-01-11

**Authors:** Rebecca M. Legere, Noah D. Cohen, Cristina Poveda, Jocelyne M. Bray, Rola Barhoumi, Joseph A. Szule, Andrés de la Concha-Bermejillo, Angela I. Bordin, Jeroen Pollet

**Affiliations:** 1 Department of Large Animal Clinical Sciences, College of Veterinary Medicine, Texas A&M University, College Station, TX USA; 2 Department of Pediatrics, National School of Tropical Medicine, Department of Pediatrics, Baylor College of Medicine, Houston, TX USA; 3 Texas Children’s Hospital Center for Vaccine Development, Baylor College of Medicine, 1102 Bates Street, Houston, TX USA; 4 Department of Veterinary Integrative Biosciences, College of Veterinary Medicine, Texas A&M University, College Station, TX USA; 5 Department of Veterinary Pathobiology, College of Veterinary Medicine, Texas A&M University, College Station, TX USA; 6 Texas A&M Veterinary Medical Diagnostic Laboratory, Texas A&M University, College Station, TX USA

**Keywords:** Immunology, Molecular biology, Medical research, Molecular medicine

## Abstract

Vaccines and therapeutics using in vitro transcribed mRNA hold enormous potential for human and veterinary medicine. Transfection agents are widely considered to be necessary to protect mRNA and enhance transfection, but they add expense and raise concerns regarding quality control and safety. We found that such complex mRNA delivery systems can be avoided when transfecting epithelial cells by aerosolizing the mRNA into micron-sized droplets. In an equine in vivo model, we demonstrated that the translation of mRNA into a functional protein did not depend on the addition of a polyethylenimine (PEI)-derived transfection agent. We were able to safely and effectively transfect the bronchial epithelium of foals using naked mRNA (i.e., mRNA formulated in a sodium citrate buffer without a delivery vehicle). Endoscopic examination of the bronchial tree and histology of mucosal biopsies indicated no gross or microscopic adverse effects of the transfection. Our data suggest that mRNA administered by an atomization device eliminates the need for chemical transfection agents, which can reduce the cost and the safety risks of delivering mRNA to the respiratory tract of animals and humans.

## Introduction

The field of mRNA vaccines and therapeutics is rapidly maturing under the pressure of the COVID-19 pandemic, with several vaccine candidates on-track to become the first mRNA-based products approved for clinical use^1,2^. Messenger RNA has gained favor as a vaccine platform because production is scalable, cell-free, and easily standardized, thereby avoiding many problems for manufacturing pertaining to purification and quality that typically slow the development of traditional protein-based technologies^3^. If proven effective and safe, the projected wide-scale distribution of mRNA-based COVID-19 vaccines will generate a wealth of data about in vitro transcribed (IVT) mRNA products. This knowledge will likely expand the application of mRNA technologies to many medical problems beyond COVID-19. Besides vaccines, mRNA has broad applicability, with enormous potential benefit for therapeutic purposes in human and veterinary medicine, including genetic, infectious, metabolic, musculoskeletal, and neoplastic diseases^3–8^. For example, in vivo delivery of mRNA-encoded antibodies is an elegant solution to produce antibody-based therapeutics with virtually unlimited options^4,9–12^.

Since the original reports of the first successful translation of IVT mRNA in mice^13,14^, much progress has been made to improve translation efficiency and overcome problems of stability. We now better understand the innate inflammatory responses to IVT mRNA, such that it can be avoided by using optimized codons and modified uracil nucleosides, and increasing the product purity^3–5,15,16^. Notwithstanding that naked mRNA (i.e., mRNA delivered without a delivery vehicle) has been applied in several in vivo studies, it has become dogma that efficient carriers (so-called transfection agents) are needed to substantially enhance mRNA stability and transfection efficiency^17,18^. A variety of vehicles have been developed to protect mRNA and to enhance the efficiency of transfection of mammalian cells, but these vehicles pose concerns for added expense, complex quality control, and safety in vivo^3,4,19^. While such packaging may indeed be needed for most systemic applications because of renal filtration of mRNA^20^ and degradation by RNAse enzymes in serum^4,21^, it is not always necessary or beneficial for certain local mRNA applications^21^. For example, IVT mRNA vaccines encoding tumor-associated antigens have been injected intranodally into patients either with advanced melanoma or with hepatocellular carcinoma^3,21^. Intra-tracheal delivery of naked IVT-mRNA has been demonstrated by different research groups in mice^10,22^, and the vaginal epithelium of sheep has been locally transfected without using a transfection vehicle^11^.

Our research is focused on applications of mRNA for respiratory diseases. Messenger RNA can be effectively delivered as an aerosol to the lungs via nebulization^23,24^. This non-invasive method of drug delivery is very promising for using mRNA for the prevention or treatment of respiratory diseases. The large surface area of the lungs allows for larger doses and much higher local concentrations of the transcribed protein compared to traditional parenteral applications^24^. We more specifically aim to transfect the airways of an equine model to deliver immuno-therapeutic and immuno-prophylactic mRNAs. Here, we demonstrate in vivo for the first time that transfection of the respiratory tract of a large animal can be done safely and effectively by aerosolizing mRNA using naked mRNA (i.e., mRNA in sodium citrate buffer diluted in water). These findings are of broad-based benefit to further investigations of clinical applications of mRNA transfection for therapy in human and veterinary medicine because they indicate that a transfection agent such as a polyethylenimine (PEI)-derivative or lipid nanoparticles might not be necessary for all modes of mRNA delivery.

## Results

### In vitro transfection of equine bronchial epithelial cells and bronchial explants with aerosolized mRNA encoding enhanced green fluorescent protein (eGFP)

**Figure 1:**
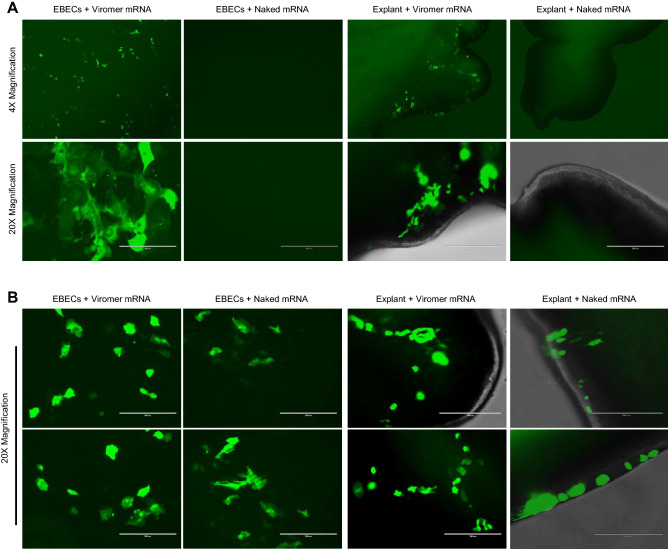
Transfection of cultured equine respiratory tissues using eGFP mRNA delivered as an aerosol of either naked mRNA or mRNA formulated with Viromer. **(A)** Transfection and eGFP expression of cultured equine bronchial epithelial cells (EBECs) and bronchial explants submerged in cell media, detected by fluorescent microscopy after 24 h incubation at 37 °C, was successful using Viromer transfection agent but failed when naked mRNA was delivered to EBECs or explants in media. The experiment was performed in duplicate, using 0.2 µg mRNA in each well of EBECs and 0.8 µg mRNA in each well of bronchial explants. Transfection with mRNA solution delivered by aerosolization was performed 48 h after establishing EBECs in culture and 72 h after establishing bronchial explant cultures. Each condition is shown at low magnification (upper row ×4, bottom row ×20 with scale bar 200 µm). All cultured tissues were derived from the same horse. **(B)** Transfection of cultured equine bronchial epithelial cells (EBECs) and bronchial explants using aerosolized mRNA expressing eGFP with media removed. Conditions were the same as in **(A)**, except that media was removed and cultured tissues were washed twice with warmed phosphate-buffered saline prior to mRNA delivery; the mRNA solutions were delivered to cultured tissues using an aerosolizer device, incubated at 37 °C for 10 min, and then culture media was returned for subsequent incubation. All cultured tissues were derived from the same horse.

5-methoxyuridine modified mRNA, encoding eGFP, was formulated either as naked mRNA or with Viromer mRNA, a commercially-available polyethylenimine (PEI)-derived transfection agent ^25,26^ that has been reported to be an effective transfection agent^10,27,28^. These mRNA formulations were administered as an aerosol to cultured equine bronchial epithelial cells (EBECs) and equine bronchial explants using an atomization device. When the EBECs and bronchial explants were submerged in cell media, successful translation of eGFP was only observed with the mRNA formulations that include Viromer (Fig. 1A). Failure to transfect with naked mRNA can be attributed to enzymatic degradation of the naked mRNA by the culture media used for the EBECs or the explants (DMEM/F12) (Supplemental Fig. 1). However, after temporarily removing the cell media, we could also successfully transfect EBECs and bronchial explants with aerosolized naked mRNA (Fig. 1B). Moreover, when mRNA was entrapped in micron-sized droplets created by the atomizer, cells were transfected as efficient and effective with naked mRNA as with the mRNA prepared with Viromer.

**Figure 2:**
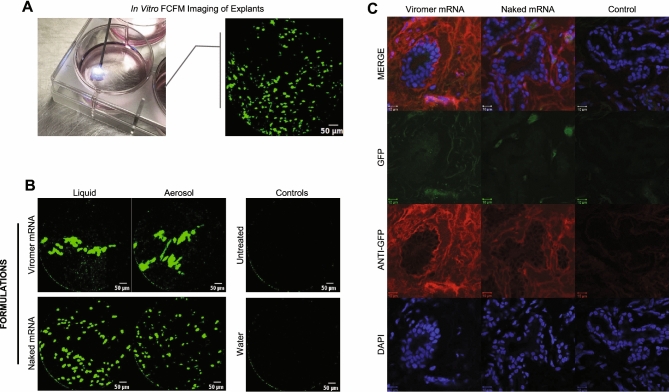
Expression of eGFP in transfected bronchial explants was achieved with naked mRNA and Viromer mRNA as detected by fiberoptic confocal fluorescent endomicroscopy (FCFM) imaging and immunohistochemistry. Cultured bronchial explants were transfected with 0.8 µg eGFP mRNA, delivered in either water or Viromer, with culture media temporarily removed. FCFM imaging in the 488 nm excitation channel was performed 24 h after transfection of the cultured bronchial explants. **(A)** Continuous 1-min video recordings were recorded of each explant. **(B)** Representative images from FCFM demonstrating eGFP expression in all transfected explants and none in the control explants. **(C)** Immunohistochemistry staining of fixed samples from the same explants from **(B)**, stained with a polyclonal anti-GFP antibody labeled with Alexa Fluor 647, confirmed eGFP expression (green) in transfected bronchial epithelium, co-localized with the anti-GFP (red) and nuclear staining (DAPI, blue). Note that the red anti-GFP does not co-localize with the eGFP because the latter was greatly diminished by tissue fixation and because fixed tissues can produce green autofluorescence indistinguishable from GFP. The purpose of this figure is to demonstrate localization of GFP in respiratory epithelial cells, indicating successful transfection.

For our in vivo experiments, we used a fluorescence-detection probe passed transendoscopically with fiberoptic confocal fluorescence microscopic (FCFM) detection system to detect green fluorescence indicating successful transfection with mRNA encoding eGFP. To bridge the results of the fluorescent images made with a standard confocal microscope (Fig. 1B), we used the fluorescence-detection probe to detect eGFP expressed in transfected explants (Fig. 2A,B). To confirm that the fluorescence detected with the FCFM system was from eGFP and not the result of autofluorescence, we used immunohistochemistry staining of fixed samples from those same explants, stained with a polyclonal anti-GFP antibody labeled with Alexa Fluor 647 that was readily distinguishable from any green autofluorescence (Fig. 2C); please note that fixation of tissues diminishes GFP signaling. Subjectively, there did not appear to be evidence of greater transfection with mRNA + Viromer than with naked mRNA in explants (Fig. 2B).

### In vivo transfection of equine guttural pouch epithelium with eGFP mRNA

**Figure 3:**
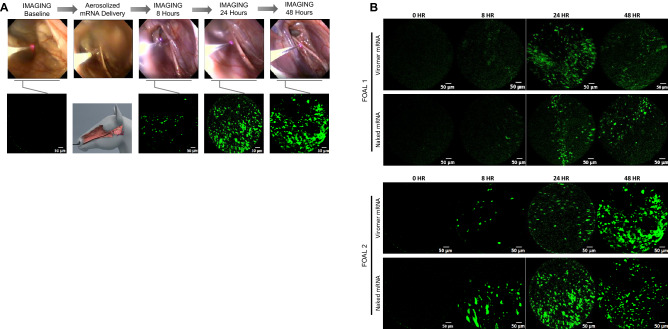
Aerosolized delivery of mRNA using either naked mRNA or mRNA with Viromer as a transfection agent results in green fluorescence of the guttural pouch epithelium detectable by transendoscopic fiberoptic confocal fluorescent microscopic (FCFM) imaging.** (A)** Schematic representation of the experiment. Endoscopic images (top row) and FCFM images (bottom row) were acquired by FCFM imaging immediately prior to transfection of the guttural pouch epithelium, and then at 8, 24, and 48 h post-transfection. Transfection was performed in each of 2 foals using 250 µg eGFP mRNA, delivered either as naked mRNA or Viromer mRNA. In the top row, the FCFM detection probe is visible as a bright horizontal line emitting light at the end in contact with the guttural pouch mucosa. In the bottom row, the second image from the left is an anatomical schematic to demonstrate the location of transendoscopic transfection and FCFM imaging. **(B)** Representative images from FCFM demonstrating minimal autofluorescence at baseline and eGFP expression at all post-transfection time-points (8, 24 and 48 h). FCFM was performed using excitation in the 488 nm channel. Please note that there was no apparent difference during real-time imaging between fluorescence in the pouches aerosolized with naked mRNA and those aerosolized with Viromer mRNA.

Next, we compared the safety and efficacy of aerosolized mRNA encoding eGFP using either the Viromer vehicle or naked mRNA in the guttural pouches of horses. The equine guttural pouches are paired diverticula (right or left side) of the Eustachian tubes (Supplemental Fig. 2), which enabled each foal to serve as its control. mRNA was delivered to in the guttural pouches of 2 foals transendoscopically using a 1.6-m, commercially-available aerosolizer passed through the biopsy channel of an endoscope (Olympus GIF 160). One guttural pouch was transfected with mRNA prepared with Viromer as a transfection vehicle and the other pouch was transfected with naked mRNA; the individual guttural pouch that was transfected with Viromer was assigned randomly by flipping a coin. The mRNA was delivered in a final volume of 2.5 ml containing 250 µg of IVT mRNA. This transendoscopic approach allowed us to see specifically where the mRNA was sprayed within the pouch (Fig. 3). Green fluorescence indicating successful transfection was detected using the FCFM detection system. A negligible amount of autofluorescence was observed in the guttural pouches of both foals at 0 h; at 8 h post-transfection, there was green fluorescence detectable in both foals, that was observed more prominently again at 24 and 48 h (Fig. 3). Adverse effects were not observed in the foals when examined endoscopically at 8, 24, and 48 h after transfection (Supplemental Fig. 3), and no adverse clinical signs were observed during the 14 days post-transfection.

### In vivo transfection of equine bronchial epithelium using naked eGFP mRNA

**Figure 4:**
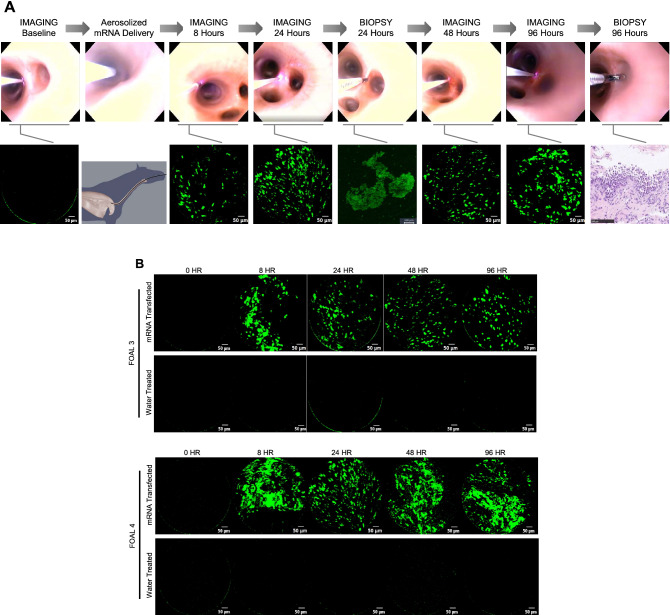
Aerosolized delivery of naked mRNA alone results in green fluorescence of the bronchial epithelium of foals detectable by transendoscopic fiberoptic confocal fluorescent microscopic (FCFM) imaging. **(A)** Schematic representation of the experiment. Endoscopic images (top row) and FCFM images (bottom row) were acquired by FCFM imaging immediately prior to transfection (or sham treatment with water only) of the bronchial epithelium, and then at 8, 24, 48, and 96 h post-transfection. At 24 h, a mucosal biopsy was obtained from both the transfected and control regions to assess eGFP expression in the epithelial tissues. At 96 h, a second mucosal biopsy was obtained from the transfected and control regions to assess microscopic evidence of mucosal inflammation secondary to transfection. In the top row, the FCFM detection probe is visible as a bright horizontal line emitting light at the end in contact with the bronchial mucosa; the mucosal biopsy instrument is similarly visible in the images labeled as biopsy. In the bottom row, the second image from the left is an anatomical schematic to demonstrate the location of transendoscopic bronchial transfection and FCFM imaging. **(B)** Representative images from FCFM demonstrating minimal autofluorescence in samples collected at baseline (0 h [hr]) and in water-treated bronchi at all time points, and eGFP expression at all post-transfection time-points (8, 24, 48, and 96 h) in each transfected bronchus. FCFM was performed using excitation in the 488 nm channel.

Because our long-term goal is to transfect the lower airways of horses with aerosolized mRNA, we next examined whether aerosolized naked mRNA could be safely and effectively used to transfect the bronchial epithelium of 2 foals. One lung (right or left) was selected a priori by coin-flip to be aerosolized with only water (control) and the other side was aerosolized with naked mRNA. A dose of 250 µg of mRNA was delivered in a volume of 1.25 ml (Foal 3) or 2.5 ml (Foal 4) using the same transendoscopic aerosolizing catheter and endoscope described above; in each foal, the control side was aerosolized with the same volume of water as the treated side. The rationale for using 2 different volumes was to examine whether dilution of mRNA in a larger volume (as will occur with nebulization) reduced the efficacy of transfection. The endoscope was passed to the carina and then passed into each mainstem (primary) bronchus. A bifurcation of a secondary bronchus was identified and probed with the FCFM system to document the absence of signal; the FCFM system was calibrated before use to improve accuracy. After completing the procedure in 1 mainstem bronchus, the process of signal detection was repeated in the mainstem bronchus of the other side of the horse. Then, we aerosolized the origin of a secondary mainstem bronchus on the side assigned to water (performed before mRNA to avoid cross-contamination via the biopsy channel of the endoscope), followed by aerosolization of naked eGFP mRNA onto the origin of a secondary mainstem bronchus of the other side of the horse. Endoscopic images were collected to allow us to map back to the site of transfection on each side of the horse. Endoscopy was repeated at 8, 24, 48, and 96 h after aerosolization in both foals. At each examination, fluorescence microscopy was performed transendoscopically using the FCFM probe, and images were recorded (Fig. 4A); we examined the water-treated (control) side first to avoid any potential carry-over/cross-contamination. At each time-point, we observed green fluorescence indicating successful transfection of eGFP on the transfected side but not on the side aerosolized with water alone (Fig. 4B).

**Figure 5:**
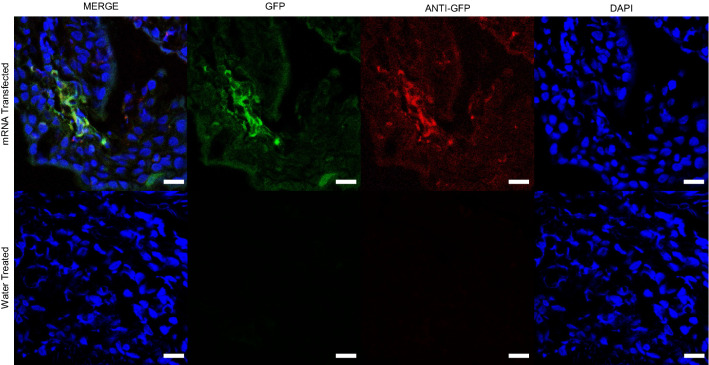
Immunohistochemical demonstration of green fluorescence in bronchial epithelium transfected with eGFP naked mRNA. Bronchial mucosal biopsies obtained 24 h after aerosolized transfection with 250 µg eGFP naked mRNA were fixed, cryosectioned, and mounted for fluorescent imaging. Immunohistochemistry staining of fixed samples stained with a polyclonal anti-GFP antibody labeled with Alexa Fluor 647, confirmed eGFP expression (green) in bronchial epithelium, co-localized with the anti-GFP (red) and nuclear staining (DAPI, blue). The merged image demonstrates that GFP was detected in the mucosal biopsy from the mRNA-transfected bronchus but not the water-treated bronchus, confirming the green fluorescence detected using FCFM. Note that the red anti-GFP does not always appear to co-localize with the eGFP because the latter was greatly diminished by tissue fixation, and because fixed tissues can produce green autofluorescence indistinguishable from GFP. The purpose of this figure is to demonstrate localization of GFP in respiratory epithelial cells, indicating successful transfection. White bars represent 10 microns.

To further substantiate transfection, we obtained transendoscopic mucosal biopsy samples at 24 h post-transfection from the areas that were aerosolized with either water alone or naked mRNA. Confocal fluorescent microscopy confirmed eGFP expression in naked mRNA transfected tissues by primary labeling of eGFP protein in epithelial cells; no GFP expression was seen in the biopsy samples of mucosa treated with water alone (Fig. 5).

**Figure 6:**
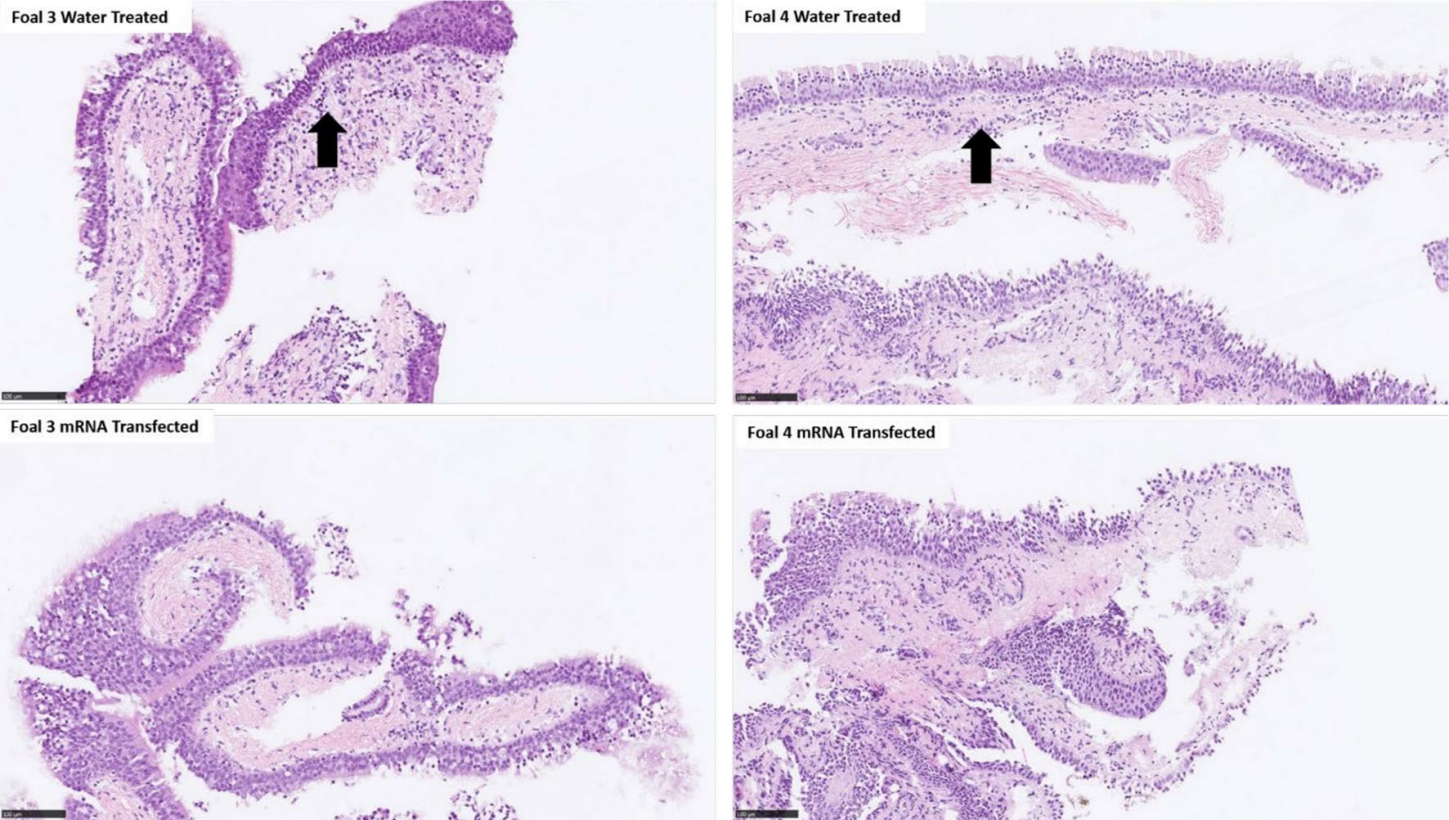
Transfection with eGFP did not result in microscopic evidence of inflammation. Minimal lymphocytic infiltration was present in the lamina propria of water-treated controls (arrows) from both foals but not in the mRNA-transfected tissues. Bronchial biopsies were obtained at 96 h from the water-treated and mRNA-transfected treated bronchial mucosa via transendoscopic smooth-mouthed biopsy instrument. Biopsies were fixed in 10% neutral phosphate-buffered formalin, embedded in paraffin, sectioned at 4 microns, and stained with hematoxylin and eosin. The scale bar is 100 µm.

Endoscopic examination demonstrated the absence of any gross changes to the aerosolized areas, and foals remained free of any clinical signs after each procedure and up to the end of the 14-day monitoring period post-transfection (Supplementary Fig. 5). To further substantiate the safety of the procedure, we collected biopsies of the aerosolized mucosa at 96 h (Fig. 6). Microscopic examination of the bronchial biopsies revealed no inflammation in mRNA-transfected samples and multifocal areas of minimal infiltration of lymphocytes in the lamina propria of the control (water-only) samples.

## Discussion

Recent technological innovations have enabled IVT mRNA to become a promising therapeutic tool with numerous medical applications^3–9^. The production and purification process of IVT mRNA can easily be standardized, thereby avoiding the need for costly product-specific production and purification steps. Additional advantages of this approach include natural protein folding and correct post-translation modifications. The use of mRNA can increase the half-life of the therapeutic proteins because mRNA is translated over a longer period of time relative to the administration of proteins such as monoclonal antibodies. Messenger RNA is a transient product and has a positive safety record with no potential risk for reverse transcription or genomic insertion. Typically IVT mRNA therapeutics are delivered systematically by intravenous (IV) administration^3–6,8^. This approach is not the most effective when a local therapeutic application is needed, and often requires large doses^4,5^.

Respiratory diseases are a leading cause of death and disability in the world^29–32^. The lung is a vital organ with a large exposed surface area, which makes it vulnerable to airborne infections such as tuberculosis, COVID-19, and influenza. However, this large contact area of the lungs can also be used as an effective entry point for the administration of mRNA by nebulization that can then be translated into proteins for local action^24^. To prevent immune activation and inflammation in the lung, we used highly purified codon-optimized IVT mRNA, which was made with 5-methoxyuridine instead of uridine^4,5,19^. Moreover, limiting any innate immune activation also increases the efficiency of protein translation^33,34^.

Delivery systems, predominately based on lipid nanoparticles^4,5,16,19^, have been developed to protect mRNA administered parenterally or topically for delivery to targeted cells both in vitro and in vivo. These transfection delivery agents are primarily designed to shield the mRNA from enzymatic degradation, increase mRNA uptake by targeted cells, and promote subsequent escape from intracellular endosomes into the cytoplasm, thereby increasing translation into the desired protein products. We selected Viromer as an mRNA delivery system because of our experience with its superior efficacy as a delivery system for parenteral mRNA-based vaccines in mice. Although such delivery systems appear to be necessary for parenteral administration, here we show for the first time that aerosolization of mRNA to the upper or lower respiratory tract of a large animal can be accomplished in vivo with naked IVT mRNA. Qualitatively, water was as efficacious as the Viromer vehicle for aerosolized mRNA transfection in foals in vitro and in vivo. The protective functions of transfection delivery agents may not be necessary for aerosolization of the respiratory tract because mRNA is applied directly to epithelial cells, thereby reducing exposure to enzymatic degradation. Our findings are important because delivering naked mRNA to the airways greatly reduces the costs and complexity of its use for treating and preventing respiratory tract diseases. As previously noted by others^11^, the use of water alone to reconstitute mRNA for aerosol delivery to mucosal membranes offers the advantages of technical simplicity and avoiding the need for a cold-chain when lyophilized mRNA is used^35^. This is one of the major concerns about the advanced COVID-19 vaccine currently in advanced development. Consequently, aerosolized mRNA-based vaccines and therapeutics could be used in low-resource environments for both human and veterinary medicine.

Naked mRNA aerosolized to EBECs or explants submerged in media failed to result in transfection (Fig. 1). Although we demonstrated that degradation of mRNA by the media caused this (Supplemental Fig. 1), we hypothesize that particle size also influences the efficacy of transfection. Whereas droplets administered into liquid media dissolve, aerosolized droplet particles will directly reach cells. Thus, the particle size of the droplets may influence the efficacy of transfection. The atomizer used in this study produces droplets of sizes between 30 to 100 µm in diameter, and these enabled safe and effective transfection in vitro and in vivo. However, due to their size, we expect that the mRNA droplets could only reach cells in close range of the tip of the aerosolizer. Particle sizes < 5 µm in diameter must be generated to reach the lower airways via nebulization ^36^. Further studies to evaluate the effectiveness and efficiency of smaller particle sizes are warranted.

Delivery of naked mRNA appeared safe. We saw no evidence of adverse effects based on physical or endoscopic examinations of foals in either the guttural pouches or bronchi, and there was no microscopic evidence of inflammation in the transfected epithelium after 96 h. The very mild inflammation infiltration of lymphocytes observed in the bronchial mucosa treated with only water of both foals was minimal and may have resulted from repeated probing of the “Water Treated” sites in an effort to ensure that fluorescence was not missed. In previous studies of aerosolized transfection of the lungs of rodents, the animals were subsequently euthanized and the treated respiratory tissues were removed en bloc for further ex vivo study^10,22^. Thus, this is the first report of in vivo evaluation of safety post-transfection without euthanasia and with several weeks of follow-up monitoring in a large animal model.

This study has a number of limitations. The sample size was very small. We only examined a single administration of a single dose of mRNA. We did not determine whether the duration of fluorescence (as long as 96 h after administration of mRNA) was due to ongoing protein production or persistence of the eGFP in cells. For future studies of biologically functional proteins, the time-course of protein expression as well as the cellular localization of protein products will need to be evaluated. We did not optimally calibrate the FCFM device for imaging Foal 1, which diminished the quality of images for this foal; however, we obtained useful images and subsequently optimized the device for use in Foal 2. We were not able to quantify whether there were differences in fluorescence between mRNA in Viromer and naked mRNA in the guttural pouches because the exact area sprayed was not visibly distinct and because it was difficult to achieve consistent contact of the probe at the optimal angle for signal detection. Nevertheless, we documented that naked mRNA appeared qualitatively to be as effective as mRNA formulated with Viromer for transfecting equine respiratory epithelial cells. Finally, our model is limited to the expression of green fluorescent protein, which may not be equivalent to the expression of a more complex protein such as an antibody, however, others have shown translation of mRNA into functional antibodies by the respiratory epithelium in rodent models^9,10,22^.

When imaged using the trans-endoscopic FCFM system and excitation with light of 488 nm wavelength, healthy human bronchial mucosa has been reported to have strong autofluorescence, and absence of this autofluorescence is indicative of bronchial pathology such as inflammation or neoplasia^37,38^. Because of this autofluorescence, alternatives to GFP have been used for respiratory tract research such as bioluminescent assays (e.g., luciferase) or scintigraphy^39^. Although we observed very minimal autofluorescence of equine respiratory mucosa using FCFM in vivo, we saw considerable green autofluorescence in bronchial explant samples from our foals, particularly after fixation as has been reported in human bronchial specimens^40^. This autofluorescence was more notable in the subepithelial connective tissue, consistent with the autofluorescence of elastin fibers of human bronchial subepithelial regions^38^. We used far-red-fluorescent anti-GFP antibodies to confirm eGFP protein expression in fixed histology samples because this autofluorescence was difficult to differentiate from true GFP signal. It is important to note that tissue fixation greatly reduces GFP signaling, as was observed in our tissue samples (Figs. 2C and 5). Nevertheless, our findings indicate that eGFP can be used in vivo to study transfection of the respiratory tract of healthy horses.

The use of mRNA for vaccines and therapeutics has enormous potential to benefit human and animal health. We report for the first time safe and effective transfection of the respiratory tract of a large animal by aerosolizing naked mRNA. These findings indicate that it is possible to reduce the costs and technical complexity of delivering mRNA to the airways for therapeutic or prophylactic purposes in people and animals.

## Materials and methods

All methods were performed in accordance with relevant guidelines and regulations for animal use and for laboratory practices including environmental health, occupational safety, and biosafety.

### Animal husbandry

Mares and foals were housed in paddocks at the College of Veterinary Medicine & Biomedical Sciences at Texas A&M University and brought into a climate-controlled building to perform procedures including endoscopic examinations, transfection, transendoscopic fluorescent microscopy, and transendoscopic bronchial mucosal biopsies. All studies were approved by the Institutional Animal Care and Use Committee (AUP# 2018-0381; 2020-0056; 2020-0079) and Infectious Biohazard Committee (IBC# 2017-105) of Texas A&M University and all facilities were approved by the American Association for Accreditation of Laboratory Animal Care (AAALAC).

### Endoscopic procedures

For endoscopic procedures, foals were sedated with xylazine (0.5 mg/kg; IV; Anased LA, Akorn Animal Health, Inc., Lake Forest, Illinois, USA) and butorphanol (0.04 mg/kg; Torbugesic, Zoetis, Parsippany, New Jersey, USA). An aseptically-prepared video-endoscope with an outer diameter of 9 mm (GIF 160, Olympus, Center Valley, Pennsylvania, USA) was passed either into the guttural pouches using a guide probe (GP-1818, Endoscopy Support Services, Brewster, New York, USA) or to an area approximately 2 cm distal to the bifurcation of the main-stem bronchi. Transendoscopic aerosolization of mRNA was performed using a transendoscopic sprayer (PW-5V-1, Olympus, Center Valley, Pennsylvania, USA) passed through the biopsy port of the endoscope. The mRNA solution (1.25 or 2.5 ml) was injected into the sprayer port and followed with 10 ml of room air to aerosolize the endoscopically-identified target in either the guttural pouch or bronchial mucosa. Endoscopic images were collected to allow us to identify the area sprayed during follow-up examinations.

Transendoscopic fiberoptic confocal fluorescence microscopy was performed using a commercial system (Cellvizio Dual Band, Mauna Kea Technologies, Paris, France). The 1.6-m fiberoptic fluorescence detection probe (ProFlex S-1500, Mauna Kea Technologies, Paris, France) was passed through the endoscope’s biopsy channel to allow contact with the respiratory mucosa of foals. The imaging was recorded on the Cellvizio Dual Band device and downloaded for subsequent processing and analysis.

Transendoscopic biopsies (approximately 2 mm × 2 mm) of the superficial bronchial mucosa were obtained using a sterilized, oval, smooth-mouthed biopsy forceps passed transendoscopically through the biopsy channel. Biopsy specimens were immediately placed into phosphate-buffered saline (Lonza, Basel, Switzerland). Prior to removing the endoscope, each biopsy site was examined for any signs of excessive hemorrhage prior to removal of the endoscope. Two weeks after the biopsy procedure, bronchoscopy was repeated to document healing of the biopsy sites. Each foal had 2 biopsy specimens collected from the area aerosolized with either naked mRNA or water alone: a sample was collected from the transfected or control side of each foal at 24 h post-transfection for microscopic evidence of eGFP protein, and a sample was collected from each foal 96 h after transfection for microscopic evaluation of evidence of any pathological changes (i.e., 2 biopsies/foal/per time-point, for 2 time-points).

### EBEC cultures—cell isolation and culture

Equine bronchial epithelial cells and bronchial explants were harvested postmortem from two horses with no history of respiratory disease. Primary cultures were established by standard methods^41–43^. For more details, please see Supplemental Methods.

### mRNA preparation and transfection

Commercially available mRNA expressing eGFP and optimized for transfection using 5-methoxyuridine base modifications (eGFP 5moU CleanCap mRNA, Cat# L-7201-1000, Lot# WOTL3842, Trilink Biotechnologies, San Diego, CA) was used. Viromer mRNA reagent (Cat# VmR-01LB-01, Lot# VmR06-04-02, OriGene Technologies, Inc., Rockville, MD) was prepared according to manufacturer’s instructions for in vitro transfections of 0.2 µg to EBECs or 0.8 µg mRNA to bronchial explants. For each aliquot of mRNA in Viromer reagent, an equivalent volume of sterile DNAse and RNAse-free distilled water (Cat# 10977-015, Lot# 1664497, Invitrogen, Carlsbad, CA) with an equivalent dose of mRNA was prepared concurrently (naked mRNA).

### In vitro transfections

Aerosol delivery of mRNA solutions was performed in vitro using a commercially-available aerosolizing device (MADgic mucosal atomization device, Cat# MAD600, Lot# 73J1900299, Teleflex, Morrisville, NC) held vertically 2 cm above the well with culture media removed. Dedicated aerosolizers were used for naked mRNA and Viromer mRNA administration to prevent unintentional cross-contamination between the 2 mRNA formulations. Transfections were performed on EBECs after 48 h in culture and bronchial explants after 72 h in culture.

In the first transfected EBEC and bronchial explant cultures from the adult horse, the aerosolizer contained either of the 2 mRNA formulations (Naked mRNA or Viromer mRNA), in addition to 200 µL of media which was mixed prior to administration as an aerosol to the culture well, followed by an additional 6 mL air to flush the aerosolizer device. The EBECs were transfected with 0.2 µg mRNA per well, and the bronchial explants were transfected with 0.8 µg mRNA per well. The media was changed in 4 h, and cultured tissues were monitored by phase-contrast microscopy with fluorescent filters (EVOS FL Cell Imaging Microscope, ThermoFisher Scientific, Waltham, MA) at 8, 24, and 48 h post-transfection.

The second set of transfections was performed on tissue cultures from another horse, with culture media removed from the wells followed by two washes with warmed phosphate buffed saline (1× PBS without Ca and Mg, Cat# 17-517Q, Lot# 0000799011, Lonza, Basel, Switzerland). The aerosolizer device contained either naked mRNA in citrate buffer or mRNA formulated with Viromer. Viromer transfections were administered in the same doses as previously performed. Naked mRNA transfection was administered as an aerosol to EBECs in two volumes (50 µL and 200 µL water) at two mRNA doses (0.2 µg and 0.8 µg). Naked mRNA was administered to bronchial explants at a single dose (0.8 µg in 200 µL), both as a liquid delivery and as an aerosol. In each of these conditions, the water and mRNA were incubated on the tissues at 37 °C in 5% CO_2_ in humidified conditions for 10 min, followed by the addition of warmed cell culture media (400 µL for EBECs and 600 µL for bronchial explants) to the wells. Water-treated control wells were also incubated with equal volumes of water to evaluate the effects of media removal and water incubation, and one set of untreated controls remained untreated. The media was changed in 4 h, and cultured tissues were monitored by phase-contrast microscopy with fluorescent filters at 8, 24, and 48 h post-transfection.

### In vivo transfections

For each guttural pouch transfection, 2 doses of 250 µg eGFP mRNA were prepared immediately prior to transfection, each in a total volume of 2.5 mL. The Viromer dose was prepared with modifications to the manufacturer’s reagent preparation, based on enhanced in vivo safety profile and transfection outcomes using an optimized saline:buffer ratio (unpublished data, Pollet et al.). Detailed instructions are in the Supplemental Methods. Concurrently, 250 µL mRNA solution (containing 250 µg mRNA) was added to 2250 µL sterile DNAse and RNAse-free distilled water (Invitrogen, Carlsbad, CA), vortexed 5 s, and incubated under equivalent conditions as the Viromer dose. Both foals were healthy 4-month-old Quarter Horse fillies and were sedated as previously described, with the endoscope passed into the guttural pouches. Each mRNA dose was administered to the caudal wall of the medial compartment of a guttural pouch from a 3-mL Luer-lock syringe connected to the transendoscopic aerosolizing catheter, followed by a 10-mL bolus of air to flush the catheter of residual mRNA solution. The head was elevated for 20 min after administration to prevent leakage of mRNA solution from the guttural pouches.

For each bronchial transfection, 1 dose of 250 µg of eGFP mRNA was prepared in sterile DNAse and RNAse-free distilled water (Invitrogen, Carlsbad, CA), to a total volume of 1250 µL for Foal 3 and 2500 µL for Foal 4, and kept on ice briefly until administration. A non-treated dose of sterile water was prepared in equivalent volume and kept under the same conditions. Both foals were healthy 4-month-old Quarter Horse colts and were sedated as previously described, with the endoscope passed approximately 2 cm beyond the carina. Each dose was administered to the bifurcation of a secondary bronchus from a 3-mL Luer-lock syringe connected to a transendoscopic aerosolizing catheter, followed by a 10-mL bolus of air to flush the catheter of residual mRNA solution.

### Nucleic acid electrophoresis

Electrophoresis was performed to observe the effects of media incubation and resultant degradation of the eGFP mRNA. Samples of 0.2 µg eGFP (0.2 µL) and 0.8 µg eGFP mRNA solution (0.8 µL) were directly added to 19.8 and 19.2 µL aliquots of media, respectively: sterile DNAse and RNAse-free distilled water (Invitrogen, Carlsbad, CA), bronchial explant culture media (DMEM/F12, supplemented with 2% Nu-Serum and antimicrobials), EBEC culture media (supplemented airway epithelial media, previously described), PBS (Lonza, Basel, Switzerland), and 0.9% saline (Braun Medical, Inc., Bethlehem, PA), and then incubated for 1 h at 37 °C. A control sample of mRNA solution was kept in 20 µL water on ice for 1 h as a positive control. All samples were electrophoresed in 1% agarose gels in 1 × TBE buffer (45 mM Tris base, 45 mM boric acid, 1 mM EDTA) and stained with ethidium bromide (10 mg/mL, OmniPur, Calbiochem, Burlington, MA). The eGFP band was measured against a 100-bp DNA ladder (Axygen, Glendale, AZ), and visualized on a ChemiDoc Touch Imaging System (Bio-Rad, Hercules, CA).

### Image analysis

#### Fiberoptic confocal fluorescent microscopy (FCFM)

Imaging was performed in vivo and in vitro using the Cellvizio Dual Band System (Mauna Kea Technologies, Paris, France), with imaging acquired using the ProFlex S-1500 fiberoptic probe at an excitation wavelength of 488 nm, a collection bandwidth of 505–700 nm, spatial resolution of 3.3 µm, and a frequency of 9 Hz. Calibration was performed prior to image acquisition. Imaging was recorded as continuous videos and observed in real-time, then analyzed later using proprietary software (ICViewer, Mauna Kea Technologies, Paris, France).

#### In vitro FCFM

The second set of cultured bronchial explants were imaged by FCFM 24 h post-transfection in vitro, immediately prior to fixation. Details of image acquisition and image analysis are in Supplemental Methods.

#### In vivo FCFM

Foals were sedated as previously described for each endoscopic procedure, and the S1500 fiberoptic cable was passed through the biopsy channel of the endoscope and directly contacted the respiratory mucosa. Continuous image acquisition was performed at each observation, and imaging of non-treated regions was performed prior to transfected regions to prevent potential contamination. Imaging of the guttural pouch mucosa primarily focused on the medial compartment at the treated sites on the caudal wall and floor. Imaging of the bronchial mucosa was focused on the treated bifurcation of secondary bronchi and surrounding mucosa.

#### Fluorescent immunohistochemistry

The first set of bronchial mucosal biopsies and the FCFM-imaged bronchial explants were fixed, sectioned, and stained with a polyclonal anti-GFP antibody labeled with Alexa Fluor 647 for fluorescent microscopy. Detailed instructions are in Supplemental Methods.

#### Microscopic pathology

The second set of bronchial mucosa biopsies were fixed in 10% neutral phosphate-buffered formalin, embedded in paraffin, sectioned at 4 microns and stained with hematoxylin and eosin in a Tissue-Tek Prisma Plus Automated Slide Stainer (Sakura Finetek, USA, Inc. Torrance, CA, USA). Stained slides were digitized using a NanoZoomer S360 Digital slide scanner (Hamamatsu Photonics, Bridgewater Township, NJ) and examined with NDP-Nanozoomer digital pathology software.

## Supplementary Information

Supplementary Figures.

Supplementary Information.
