# Supplementary material for: Safe and effective aerosolization of in vitro transcribed mRNA to the respiratory tract epithelium of horses without a transfection agent
Source: Sci Rep. 2021 Jan 11;11:371. doi: 10.1038/s41598-020-79855-1 (PMC7801524; doi:10.1038/s41598-020-79855-1)

Safe and effective aerosolization of *in vitro* transcribed mRNA to the respiratory tract epithelium of horses without a transfection agent

Rebecca M. Legere[1], Noah D. Cohen*[1], Cristina Poveda[2], Jocelyne M. Bray[1], Rola Barhoumi[4], Joseph A. Szule[5], Andrés de la Concha-Bermejillo[6], Angela I. Bordin[1], Jeroen Pollet*[2,3]

*Co-corresponding authors: ncohen@cvm.tamu.edu and Jeroen.pollet@bcm.edu

[1] Department of Large Animal Clinical Sciences, College of Veterinary Medicine, Texas A&M University, College Station, TX

[2] Department of Pediatrics, National School of Tropical Medicine, Department of Pediatrics, Baylor College of Medicine, Houston, TX

[3] Texas Children's Hospital Center for Vaccine Development, Baylor College of Medicine, 1102 Bates Street, Houston, TX

[4] Department of Veterinary Integrative Biosciences, College of Veterinary Medicine, Texas A&M University, College Station, TX

[5] Department of Veterinary Pathobiology, College of Veterinary Medicine, Texas A&M University, College Station, TX

[6] Texas A&M Veterinary Medical Diagnostic Laboratory, Texas A&M University, College Station, TX

Supplemental Figure 1

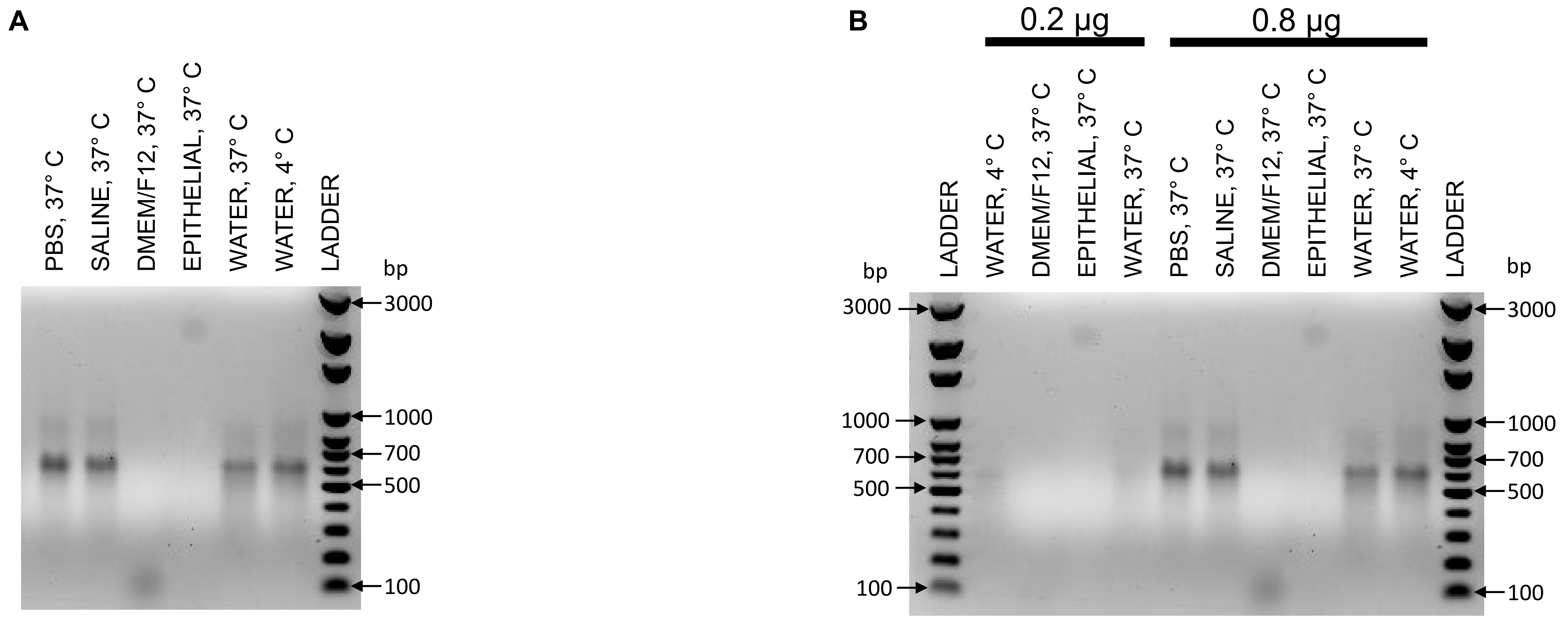

Supplemental Figure 2

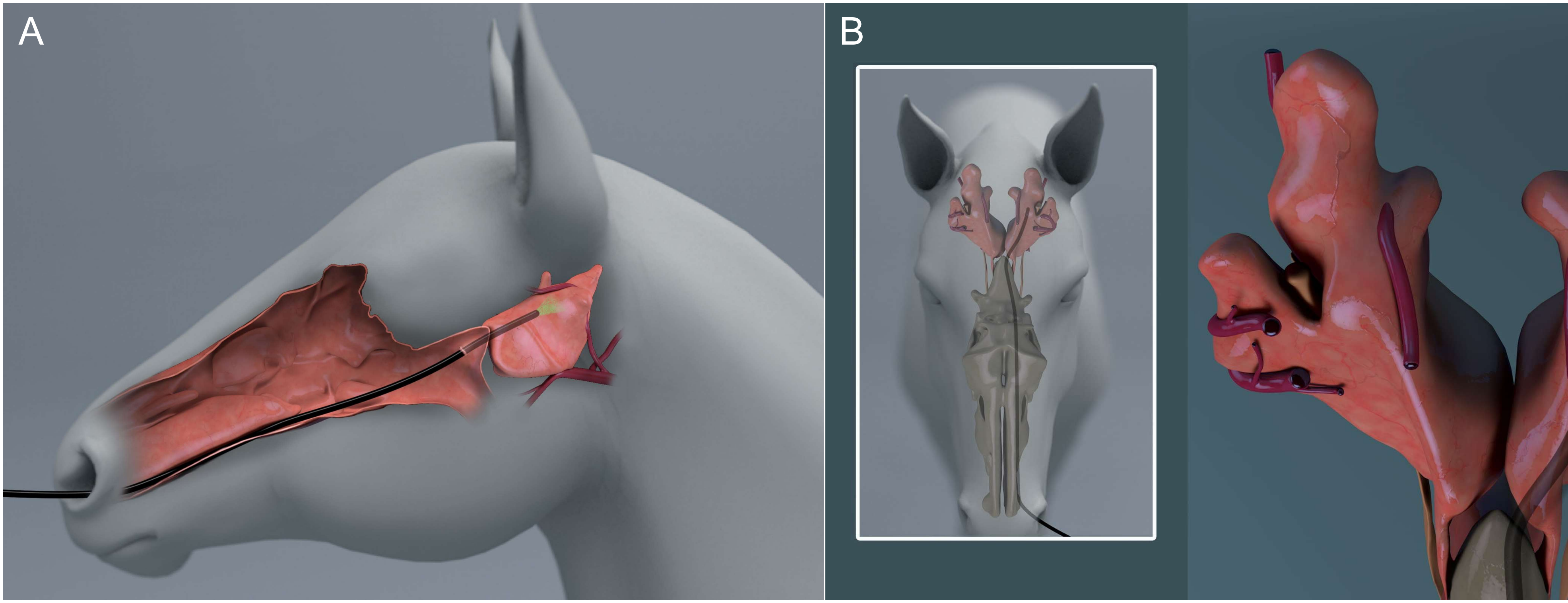

Supplemental Figure 3

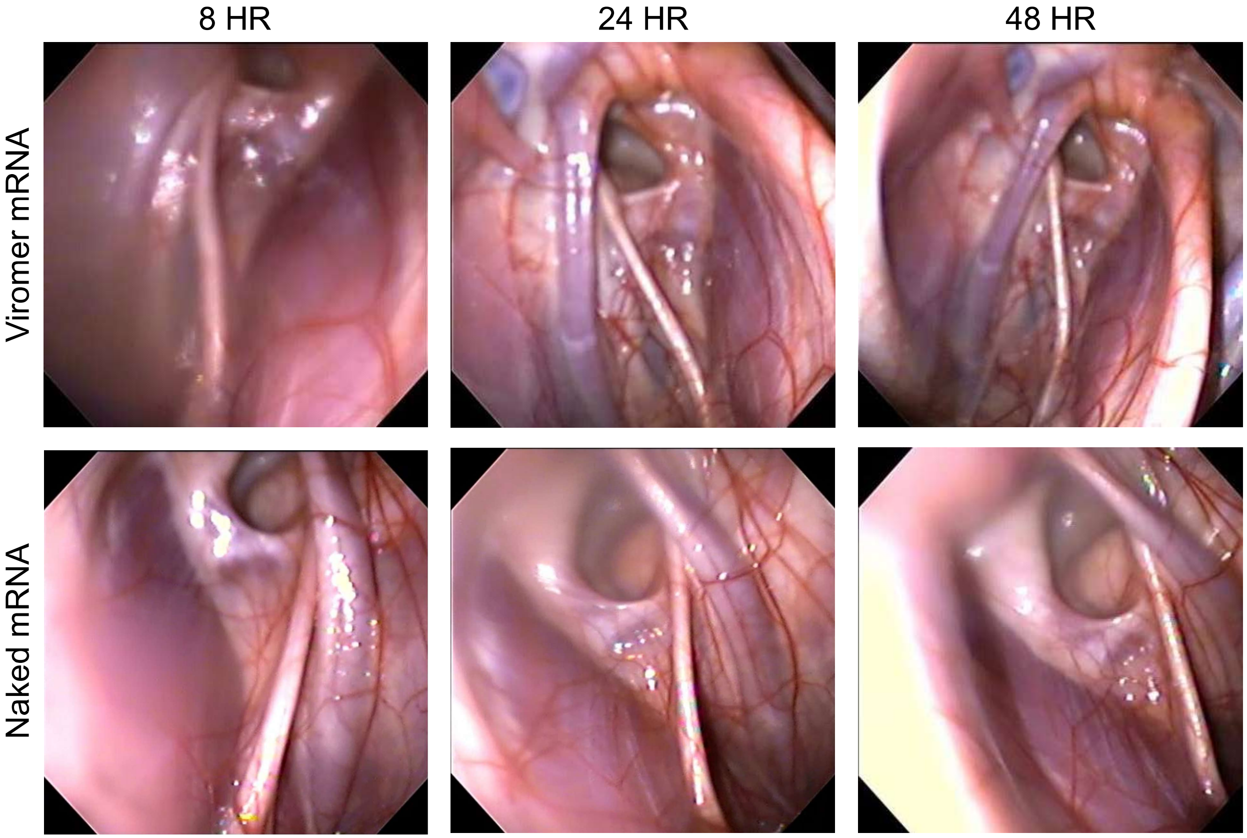

Supplemental Figure 4

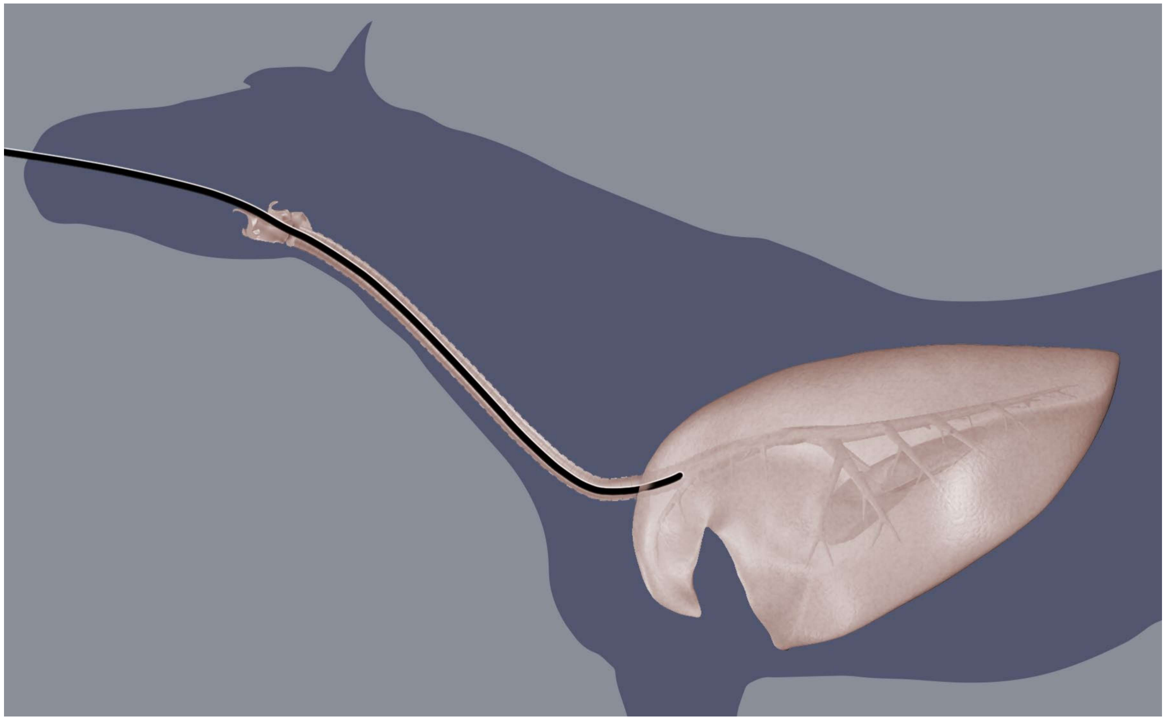

Supplemental Figure 5

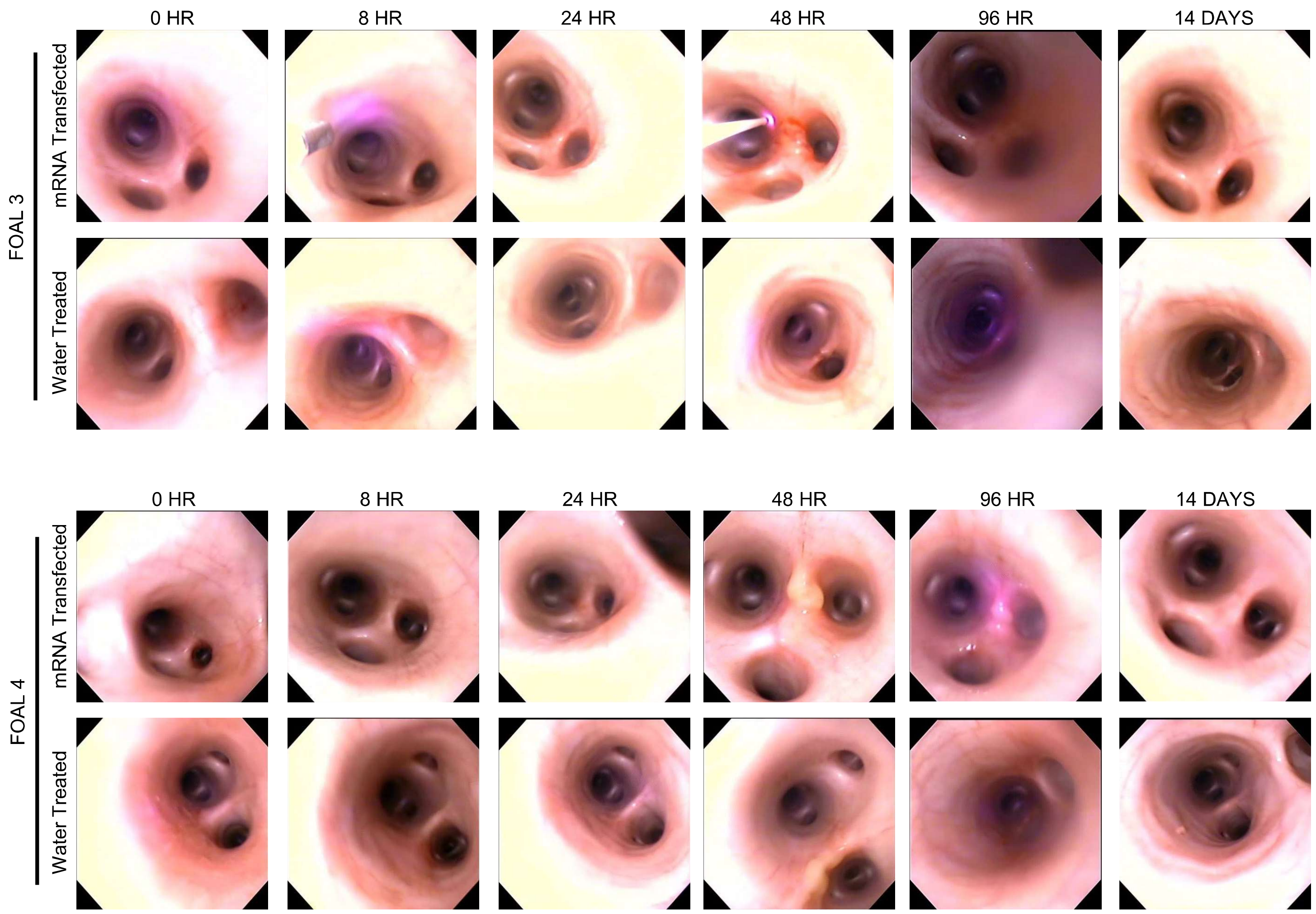

Supplement: Supplementary file 1 — Supplementary Figures. [file 41598_2020_79855_MOESM1_ESM.pdf]
