# Supplementary material for: Safe and effective aerosolization of in vitro transcribed mRNA to the respiratory tract epithelium of horses without a transfection agent
Source: Sci Rep. 2021 Jan 11;11:371. doi: 10.1038/s41598-020-79855-1 (PMC7801524; doi:10.1038/s41598-020-79855-1)
Supplement: Supplementary file 2 — Supplementary Information. [file 41598_2020_79855_MOESM2_ESM.docx]

**Supplemental Methods**

***EBEC cultures*** *–* ***cell isolation and culture***

Equine bronchial epithelial cells and bronchial explants were harvested postmortem from 2 horses with no history of respiratory disease. The lungs were removed en bloc within 1 hour of euthanasia, infused with 1 liter of ice-cold Hank’s buffered saline solution (HBSS) with penicillin (200 U/mL), streptomycin (200 µg/mL), and amphotericin B (2.5 µg/mL) into the trachea, and transported to the laboratory on ice. Lung parenchyma was bluntly removed to isolate primary and secondary bronchi, which were then sectioned in roughly 5-cm tubular segments, and submerged for 30 minutes in ice-cold HBSS with penicillin (200 U/mL), streptomycin (200 µg/mL), and amphotericin B (2.5 µg/mL). Each segment was removed, opened longitudinally, and rinsed with ice-cold HBSS. From each segment, a small bronchial epithelial explant (3 mm x 3 mm) was isolated, rinsed twice with ice-cold DMEM/F12 media (Cat# SH30271.01, Lot# AF29500570, Hyclone Laboratories, Logan, UT), and then placed into a collagen-coated 6-well plate with 0.5 mL DMEM/F12 media supplemented with 2% Nu-Serum growth medium (Cat# 355100, Corning Inc., Corning, NY), Penicillin (100 U/mL), streptomycin (100 µg/mL), and amphotericin B (0.25 µg/mL). Explants were incubated at 37°C in 5% CO_2_ with minimum disturbance to promote adherence to the plate.

The remainder of the bronchial epithelium was sharply dissected from the underlying submucosa and collected, then manually minced into approximately 1-mm segments and divided into batches of roughly 500 mg minced tissue. Each batch of tissue was placed into a Petri dish with 12 mL of 0.25% trypsin – 0.6 mM EDTA solution (Gibco, Dublin, Ireland) and incubated at 37° C for 2 hr in 5% CO_2_ under gentle agitation. Digestion was stopped by adding 5 mL ice-cold 20% fetal bovine serum (FBS) in HBSS. The suspension was filtered first through sterile double-layered gauze, then a sterile cell strainer (pore size 40 µm), both times rinsing twice with 5 mL ice-cold HBSS. The cellular suspension was centrifuged at 200 x g (5810R, Eppendorf AG, Hamburg, Germany) for 10 minutes at 4°C, the supernatant was discarded, and cells resuspended in 12 mL warmed supplemented airway culture media [Promocell Airway Epithelial Cell Growth Basal Media (Cat# C-21260, Lot# 457M245, Heidelberg, Germany), supplemented with Promocell Growth Medium SupplementPack (Cat# C-39160, Lot#454M008, Heidelberg, Germany), 10% FBS, penicillin (200 U/mL), streptomycin (200 µg/mL), and amphotericin B (2.5 µg/mL)]. This suspension was placed into a treated T75 Erlenmeyer flask and incubated for 30 minutes at 37°C in a humidified, 5% CO_2_ environment. The media was gently removed from the T75 flasks, allowing adhered fibroblasts to remain within the flask via differential plating, and the cellular suspension containing EBECs was centrifuged at 200 x g (5810R, Eppendorf AG, Hamburg, Germany) for 10 minutes at 4°C. The supernatant was discarded and cells were resuspended in 1 mL of supplemented airway culture media. Cells were counted using a cell counter (CellometerAuto T4, Nexelom Bioscience, Lawrence, MA) with trypan blue (Sigma Aldrich, St. Louis, MO) for viability assessment, and plated at a concentration of 2.5 x 10^5^ live cells/ml on a collagen-coated tissue-treated 24-well plate. Media was changed at 24 hours on all cultures, and then every 2 days afterward.

***Viromer preparation protocols for mRNA transfections***

For *in vitro* transfections, Viromer mRNA reagent (Cat# VmR-01LB-01, Lot# VmR06-04-02, OriGene Technologies, Inc., Rockville, MD) was prepared according to manufacturer’s instructions for transfections of 0.2 µg to EBECs or 0.8 µg mRNA to bronchial explants. An aliquot of the Viromer reagent (either 0.2 µL or 0.8 µL) was diluted in the provided dilution buffer (9.8 µL or 19.2 µL, respectively), vortexed for 3 to 5 seconds, then incubated at room temperature for 10 minutes. The appropriate dose of mRNA (0.2 µg or 0.8 µg) was then diluted in a separate aliquot of dilution buffer (45 µL or 180 µL, respectively), vortexed for 3 to 5 seconds, then incubated at room temperature for 5 minutes. Finally, the mRNA solution was added to the Viromer solution, vortexed, and incubated at room temperature for 15 minutes, prior to dispensing into the culture wells.

For *in vivo* transfections of the guttural pouch, Viromer mRNA reagent (Cat# VmR-01LB-01, Lot# VmR06-04-02, OriGene Technologies, Inc., Rockville, MD) was prepared with modifications to the manufacturer’s reagent preparation, based on enhanced *in vivo* safety profile and transfection outcomes using an optimized saline:buffer ratio. First, 50 µL Viromer and 1200 µL buffer were mixed and incubated for 10 minutes at room temperature, and the 250 µL mRNA solution (containing 250 µg) was incubated in 1000 µL sterile 0.9% saline (Cat#L8001, Lot# J0A033, Braun Medical, Inc., Bethlehem, PA) for 5 minutes. The saline-mRNA solution was added to the Viromer-buffer solution, vortexed for 5 seconds, and incubated for 15 minutes at room temperature, then stored on ice briefly before being administered to the foal.

***Fiberoptic confocal fluorescent microscopy (FCFM) imaging***

***In vitro FCFM:*** The second set of cultured bronchial explants were imaged by FCFM 24 hours post-transfection *in vitro*, immediately prior to fixation. Explants were individually held in place with sterile forceps, and 1-minute videos were obtained from making direct contact with the S-1500 fiberoptic probe to the apical surface of the explant and performing grid-like scanning of the explant. Following imaging, the explant was removed from culture and placed immediately into a fixing agent for immunohistochemical analysis of the tissues.

***Immunohistochemical staining***

Bronchial mucosal biopsies and the FCFM-imaged bronchial explants were fixed in a 10% paraformaldehyde and 10% sucrose solution in PBS at 4°C for 24 hours. Large pieces of tissue were first sliced less than 3 mm thick with a razor blade. Tissues, rinsed with PBS (pH 7.4) and blotted with filter paper, were placed in a mold with embedding medium (TissueTek O.C.T., Sakura Finetek, Torrance, CA) and quick-frozen on a metal block cooled with liquid nitrogen. Tissue sections were cut at 10 µm thickness with a Thermo Scientific CryoStar NX70 cryostat and mounted on microscope slides (Fisherbrand Superfrost Plus slides). One untreated biopsy section and 1 untreated bronchial explant remained in PBS (Lonza, Basel, Switzerland) and unfixed prior to cryosectioning to evaluate autofluorescence. All tissues were first evaluated for baseline autofluorescence in the 488 nm and 633 nm excitation channels (Zeiss LSM 780 NLO Multiphoton Microscope), which revealed a relatively high background signal in control tissues. Tissues were thawed for 10 minutes prior to staining, and a barrier was drawn on the slide with a hydrophobic pen. Tissues were permeabilized with 0.4% Triton X-100 (Cat# T-8787, Lot 126H2601, Sigma Aldrich, St. Louis, MO) in 1% PBS-Tween (Tween 20, Cat# 0777-1L, Lot 1855C485, Amresco Inc., Solon, OH) for 30 minutes at room temperature. Non-specific binding was blocked by incubating the tissues with 2% bovine serum albumin (Cat# A-7030, Lot 85H0269, Sigma Aldrich, St. Louis, MO) in 1% PBST for 30 minutes at room temperature. Tissues were then washed twice with 1% PBST and were stained with a polyclonal anti-GFP antibody labeled with Alexa Fluor 647 (1:200 dilution in PBST, Cat# A-31852, Invitrogen, Carlsbad, CA) for 1 hour at room temperature, followed by 2 washes with PBST. Tissues were treated with 1 drop of antifade mounting medium containing 4′,6-diamidino-2-phenylindole (DAPI) nuclear counterstain (Prolong Gold Antifade Reagent with DAPI, Invitrogen) and covered with a micro coverslip (Cat# 48393 2410, VWR). Several areas from the tissue were selected and imaged using laser excitation of 405nm, 488nm, and 633nm. Emission was collected using 448 +/- 30 nm, 525 +/- 30 nm, and above 660 nm wavelengths. Images were collected using a Zeiss LSM 780 Inverted META Confocal Microscope and were analyzed using Zen Black software (Carl Zeiss Microscopy, Oberkochen, Germany).

***Supplemental Figure 1.*** Electrophoresis of culture media and mRNA, incubated under varying conditions, demonstrated degeneration of mRNA in culture media but not in water or saline. eGFP mRNA (0.8 µg) in 20-µL aliquots of solutions of phosphate-buffered saline (PBS), 0.9% saline for injection, DMEM/F12, epithelial media (EPITHELIAL), or culture-grade water were incubated at 37°C or kept on ice (4oC) for 1 hr (water only), and then electrophoresed. The aliquots of water, 0.9% saline, and phosphate-buffered saline were sterile and cell-culture grade filtered. The DMEM/F12 media was supplemented for use in bronchial explant cultures with antimicrobials and a serum replacement solution. The epithelial media was supplemented for equine bronchial epithelial cell culture and contained multiple hormones and growth factors, antimicrobials, and fetal bovine serum. Degradation of mRNA was attributed to RNAse activity from the serum added to the culture media.

***A.*** Cropped gel.

***B.*** Original, uncropped gel (per journal requirement – this figure can be deleted).

***Supplemental Figure 2.* The guttural pouches are paired diverticula of the Eustachian tubes that open into the nasopharynx; they are unique to horses and closely related species** (*e.g.*, rhinoceri).

Schematic anatomical representation of the guttural pouches from a lateral (A) and dorsal (B) perspectives. The endoscope (represented as black linear structure) is introduced via the nares through the nasal passages and introduced into the guttural pouch for visualizing and trans-endoscopic transfection. Navigation of the endoscope within the guttural pouch permitted examination of the mucosal surface and facilitated trans-endoscopic aerosol administration of mRNA into the dorsal aspect of the medial compartment of each guttural pouch (B).

***Supplemental Figure 3.* Repeated endoscopic examinations demonstrated no adverse effects of aerosolized transfection or transfection vehicle on guttural pouch mucosa of foals.**  Representative images from similar regions of the Viromer-treated and naked mRNA-treated guttural pouches of Foal 2; results were similar for Foal 1. No abnormalities of the guttural pouch mucosa were detected in both pouches of both foals.

***Supplemental Figure 4.*** **Anatomic location of bronchial endoscopic procedures in study foals.** Schematic representation of the anatomic location of endoscopic examination and trans-endoscopic bronchial aerosolization with either naked mRNA or water only (control) and trans-endoscopic mucosal biopsy.

***Supplemental Figure 5.* Repeated endoscopic examinations demonstrated no apparent adverse effects of aerosolized transfection on respiratory mucosa in the bronchus of foals.** Representative images selected from bronchoscopy performed on Foal 3 and Foal 4 at each time-point (0, 8, 24, 48, and 96 hours [HR], and 14 days). Biopsy sites were selected on the basis of strong GFP expression in the mRNA transfected mucosa based on TCFM imaging. Biopsies were obtained at 24 hr to assess eGFP expression, and at 96 hr for microscopic pathology. Biopsy sites exhibited expected transient, focal post-sampling hyperemia, and mucous in the days following initial sampling, and they appeared healed at 14 days.
